# mtDNA deletions responsible for unsuccessful pregnancy after in- vitro fertilization

**DOI:** 10.18502/ijrm.v13i7.7373

**Published:** 2020-07-22

##  Dear Editor,

With interest we read the article by Mirabutalebi and colleagues about a study of 52 females in whom two consecutive trials of in vitro fertilizations failed to result in a successful pregnancy (1). Infertility in these females was attributed to a 4977 basepair (bp) mtDNA deletion in blood lymphocytes in 58% of the females > 35 yr of age and in 39% of the females < 35 yr of age (1). We have the following comments and concerns:

The main shortcoming of the study is that the genetic tests were carried out on lymphocytes and not on oocytes, which can be regarded as the cell in which impaired mitochondrial functions are expected. Since oocytes were available from these females, the study should be repeated in oocytes to see if the amount of deleted mtDNA was higher than in lymphocytes, as one would expect.

A further shortcoming of the study is that the heteroplasmy rate of the deletion was not determined. Carrying an mtDNA deletion alone does not imply pathology but it is the relation between non-deleted mtDNA (wild-type) and the deleted mtDNA (heteroplasmy) that is crucial for the pathogenicity of an mtDNA mutation (2). Since the copy number of mtDNA molecules in oocytes has not been systematically investigated (3) and since it is unknown which heteroplasmy rate (threshold) in oocytes is regarded to be associated with disease, we should be informed about the heteroplasmy rates of the deletion in oocytes from the 52 investigated females.

Furthermore, biochemical, functional, or cybrid studies to confirm the pathogenicity of the mtDNA deletion are missing. The deletion can be made responsible for infertility only if the pathogenicity of the variant is confirmed by functional tests. We should thus be informed if mitochondria in lymphocytes, oocytes, or other tissues showed morphological abnormalities on ultrastructural investigations in those females who carried the mutation.

We also should be informed about the number of probands who finally got pregnant after further in vitro fertilization trials and how many of these gave birth to a child with a mitochondrial disorder (MID).

The 4977 bp deletion has been previously reported in association with disease phenotypes such as hepatocellular carcinoma (4), peptic ulcer (5), astheno-zoospermia and oligo-astheno-terato-zoospermia (6), colorectal cancer (7), breast cancer (8), or infertility with varicocele (9). Thus, we should know if any of these conditions were found

in any of the 52 included females or their first-degree relatives. It is also crucial to know if the family history was positive for MID, as 4% of the mtDNA deletions are inherited from the mother's side (10).

Overall, this interesting study could be more meaningful if oocytes would have been investigated, the heteroplasmy rates of the mtDNA deletion would have been determined, the family history of the 52 included females with infertility would have been provided, and the 52 females would have been investigated clinically by an expert for MIDs regarding the presence or absence of clinical phenotypic features typical for a MID.

**Josef Finsterer${}^{1}$ M.D., Ph.D., Fulvio Alexandre Scorza${}^{2}$ M.D., Carla Alessandra Scorza${}^{2}$ M.D., Ana Claudia Fiorini${}^{3}$ M.D.**

${}^{1}$Krankenanstalt Rudolfstiftung, Messerli Institute, Vienna, Austria.

${}^{2}$Disciplina de Neurociência, Universidade Federal de São Paulo/Escola Paulista de Medicina (UNIFESP/EPM), São Paulo, Brasil.

${}^{3}$Programa de Estudos Pós-Graduado em Fonoaudiologia, Pontifícia Universidade Católica de São Paulo (PUC-SP), Brazil; Departamento de Fonoaudiologia, Escola Paulista de Medicina/Universidade Federal de São Paulo (EPM/UNIFESP), São Paulo, Brazil.

## References

[1] Sh Mirabutalebi, N Karami, Ashrafzadeh, Z Akhvansales, M Tavakoli, N Ghasemi

[2] Zhang Shi, Tong An-Li, Zhang Yun, Nie Min, Li Yu-Xiu, Wang Heng (2009). Heteroplasmy Level of the Mitochondrial tRNALeu(UUR) A3243G Mutation in a Chinese Family Is Positively Associated with Earlier Age-of-onset and Increasing Severity of Diabetes. Chinese Medical Sciences Journal.

[3] Milani Liliana, Ghiselli Fabrizio (2015). Mitochondrial activity in gametes and transmission of viable mtDNA. Biology Direct.

[4] Guo Zs, Jin Cl, Yao Zj, Wang Ym, Xu Bt (2017). Analysis of the mitochondrial 4977 bp deletion in patients with hepatocellular carcinoma. Balkan Journal of Medical Genetics.

[5] Salehi Z., Haghighi A., Haghighi S., Aminian K., Asl S. F., Mashayekhi F. (2017). Mitochondrial DNA deletion Δ4977 in peptic ulcer disease. Molecular Biology.

[6] Pal Asokek, Ambulkar Prafullas, Chuadhari Ajayr (2016). Association of large scale 4977-bp "common" deletions in sperm mitochondrial DNA with asthenozoospermia and oligoasthenoteratozoospermia. Journal of Human Reproductive Sciences.

[7] Li Tao, Chen Gui-Lan, Lan Huan, Mao Liang, Zeng Bo (2015). Prevalence of the 4977-bp and 4408-bp mitochondrial DNA deletions in mesenteric arteries from patients with colorectal cancer. Mitochondrial DNA Part A.

[8] Dimberg Jan, Hong Thai Trinh, Nguyen Linh Tu Thi, Skarstedt Marita, Löfgren Sture, Matussek Andreas (2015). Common 4977 bp deletion and novel alterations in mitochondrial DNA in Vietnamese patients with breast cancer. SpringerPlus.

[9] Gashti N. G., Salehi Z., Madani A. H., Dalivandan S. T. (2013). 4977-bp mitochondrial DNA deletion in infertile patients with varicocele. Andrologia.

[10] Poulton Joanna, Finsterer Josef, Yu-Wai-Man Patrick (2017). Genetic Counselling for Maternally Inherited Mitochondrial Disorders. Molecular Diagnosis & Therapy.

